# Supplementary material for: Combined Free-Energy Calculation and Machine Learning Methods for Understanding Ligand Unbinding Kinetics
Source: J Chem Theory Comput. 2022 Feb 23;18(4):2543–55. doi: 10.1021/acs.jctc.1c00924 (PMC9097281; doi:10.1021/acs.jctc.1c00924)
Supplement: Supplementary file 1 — ct1c00924_si_001.pdf [file ct1c00924_si_001.pdf]

Supporting Information

Combined free energy calculation and machine learning methods for understanding ligand unbinding kinetics

Magd Badaoui,[1,2‡] Pedro J Buigues,[2‡] Dénes Berta,[2] Gaurav M. Mandana,[1] Hankang Gu[2], Tamás Földes,[2] Callum J Dickson,[3] Viktor Hornak,[3] Mitsunori Kato,[3] Carla Molteni,[4] Simon D. Parsons,[5] Edina Rosta[1, 2]

[1] Department of Chemistry, King's College London, SE1 1DB London, United Kingdom
[2] Department of Physics and Astronomy, University College London, WC1E 6BT London, United Kingdom
[3] Computer-Aided Drug Discovery, Global Discovery Chemistry, Novartis Institutes for BioMedical Research, 181 Massachusetts Avenue, Cambridge, Massachusetts 02139, USA
[4] Department of Physics, King's College London, WC2R 2LS London, United Kingdom
[5] Department of Informatics, King's College London, WC2R 2LS London, United Kingdom
‡ Equal contributions

Contents

1. MD Simulations Details2
2. Atom Clustering3
3. Unbinding Collective Variables4
4. MLTSA flowchart7
5. Classification and Input Features for Machine Learning8
6. ML Training Results and Feature Analysis14
7. Free Energy Profiles19
8. 60K/4FKU system20
9. Validation of the ML Analysis23
10. Gradient Boosting Decision Trees25
11. Additional resources27
12. References27

1. MD Simulations Details

The initial atom coordinates for the three systems were built using high resolution crystal structures with the following PDB codes: **3SW4** (Resolution=1.7 Å), **4FKU** (Resolution=1.47 Å)**,** and **4FKW** (Resolution=1.8 Å)**. We present the results for 4KFU in the SI Section 8.** The systems were modelled using the AMBER ff14SB force field,[1] and the ligands using the general Amber force field (GAFF).[2] The ligand's atomic partial charges were obtained using density functional theory (DFT) ωB97X-D/def2TZVPP[3,4] as implemented in Gaussian 09 Revision E.[5] The full system was solvated with 12,000-14,000 TIP3P water molecules. Na^+ and Cl^- ions were added to neutralize the system and set a salt concentration of 0.14 M. All the MD simulations were performed using NAMD 2.12.[6]

The three systems were first minimized using a standard protocol via the steepest descent algorithm for a total of 150,000 steps followed by the equilibration of the restrained protein (1 kcal mol^{-1} $Å^{-2}$ force applied to each heavy atom of the protein) for 10 ns in NVT ensemble at 300 K via a standard MD procedure. All the production runs were performed with the NPT ensemble with a time step of 2 fs. Pressure was maintained at 1 atm by a Nosé–Hoover Langevin piston.[7] Temperature was maintained at 298 K using Langevin dynamics with a damping coefficient γ of 0.5 ps^{-1} applied to all atoms. SHAKE[8] was applied to all bonds involving hydrogen and nonbonded interactions were calculated with a cutoff of 12 Å, and a switching distance of 10 Å. The particle mesh Ewald method was used for long-range electrostatic calculations with a grid density of >1 $Å^{-3}$.[9]

An initial unbiased simulation of 20 ns was performed for each ligand. This initial simulation allows the system to equilibrate and gives us an initial trajectory to calculate the first CVs.

2. Atom Clustering

Residues with atoms that have a rotational degree of freedom with multiple equivalent positions are clustered together. During the unbinding process, if a new contact is found with one atom belonging to the cluster, then the harmonic restraint will be applied to the centre of mass of the selected clustered atoms. The use of clustered atoms reduces the fluctuation caused by the rotation of such bonds, affecting the overall harmonic restraint.

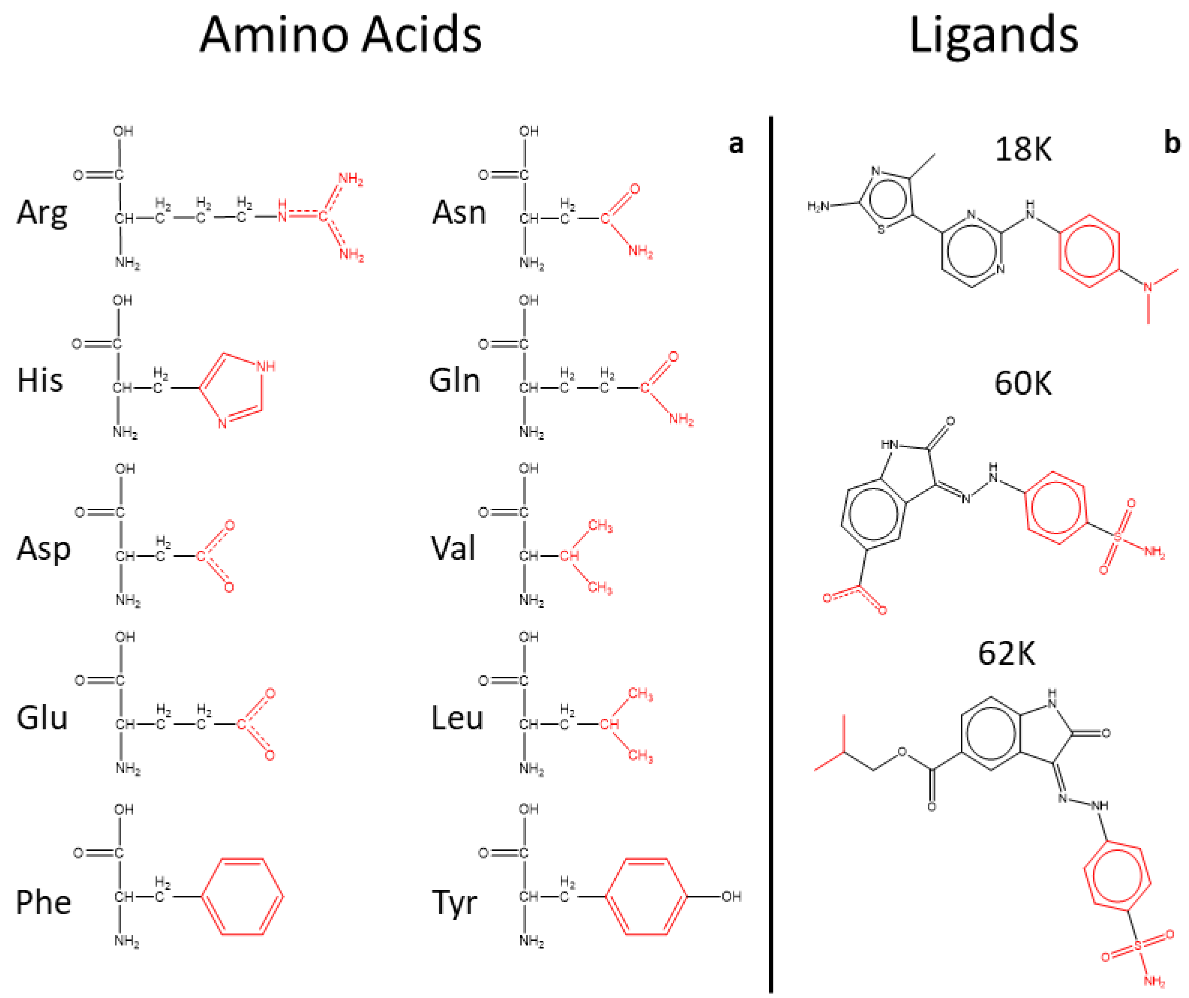

Figure S2. Chemical structures of the residues with clustered atoms, highlighted in red; a for the amino acids and b for the ligands.

3. Unbinding Collective Variables

Distances are included and excluded throughout the entire unbinding trajectory. The initial distances are calculated from the initial unbiased ligand-bound MD simulations. At every 10 ns, the biased simulation is stopped and analyzed. New close interactions between the protein and ligand will be added as part of the main CV that is biased, and previous CVs where the values exceeded the cut-off are excluded. Fig. S2.I-S2.VI display the distances that are part of the main CV along the unbinding trajectories for each ligand and each replica.

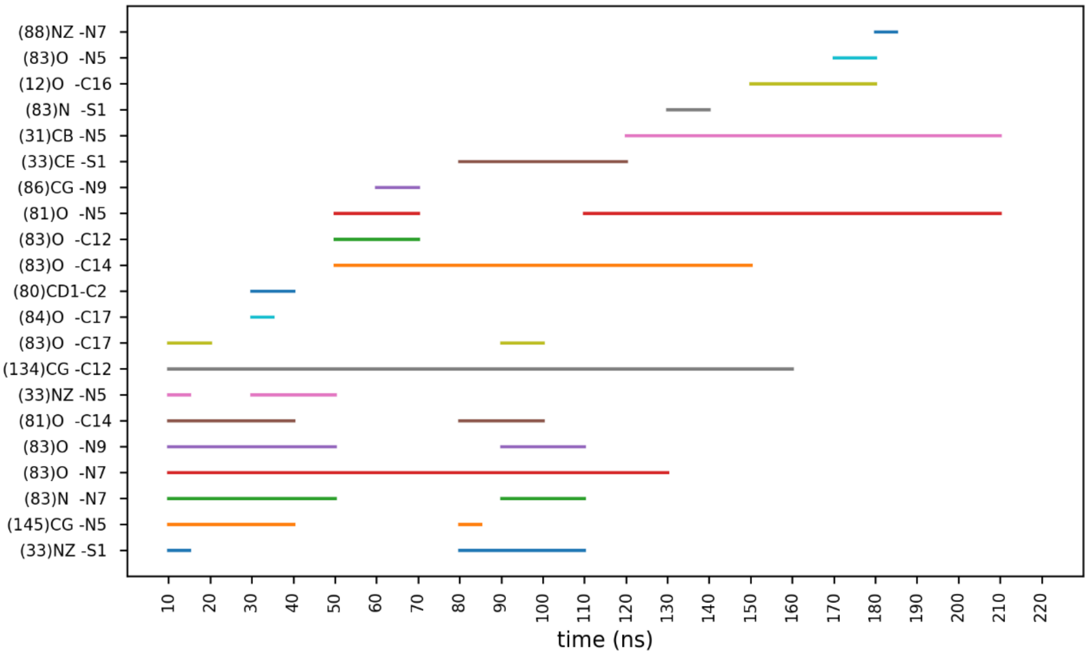

Figure S3.I. Distances used for the main CV along the trajectory for 18K replica 1.

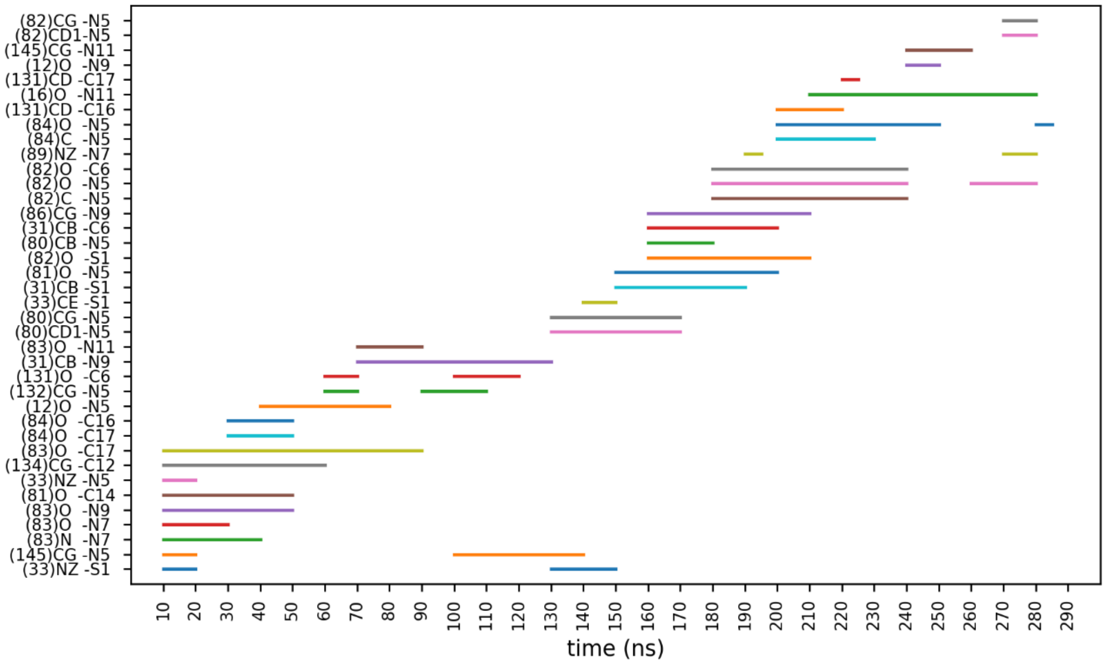

Figure S3.II. Distances used for the main CV along the trajectory for 18K replica 2.

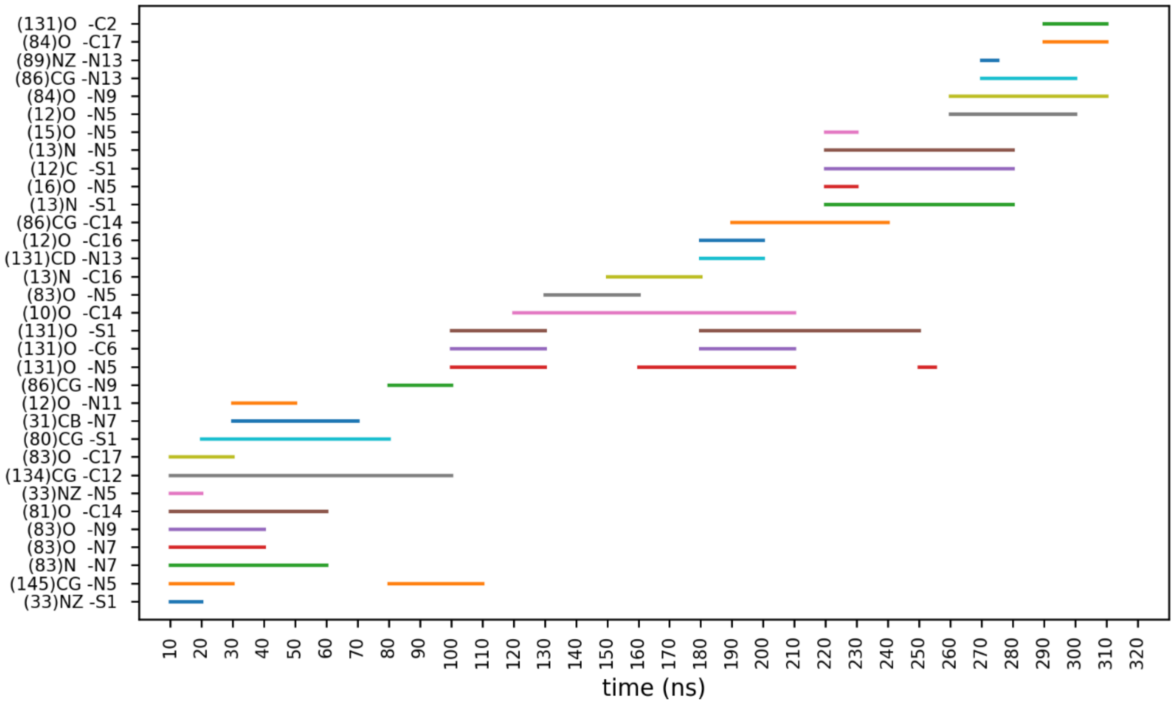

Figure S3.III. Distances used for the main CV along the trajectory 18K replica 3.

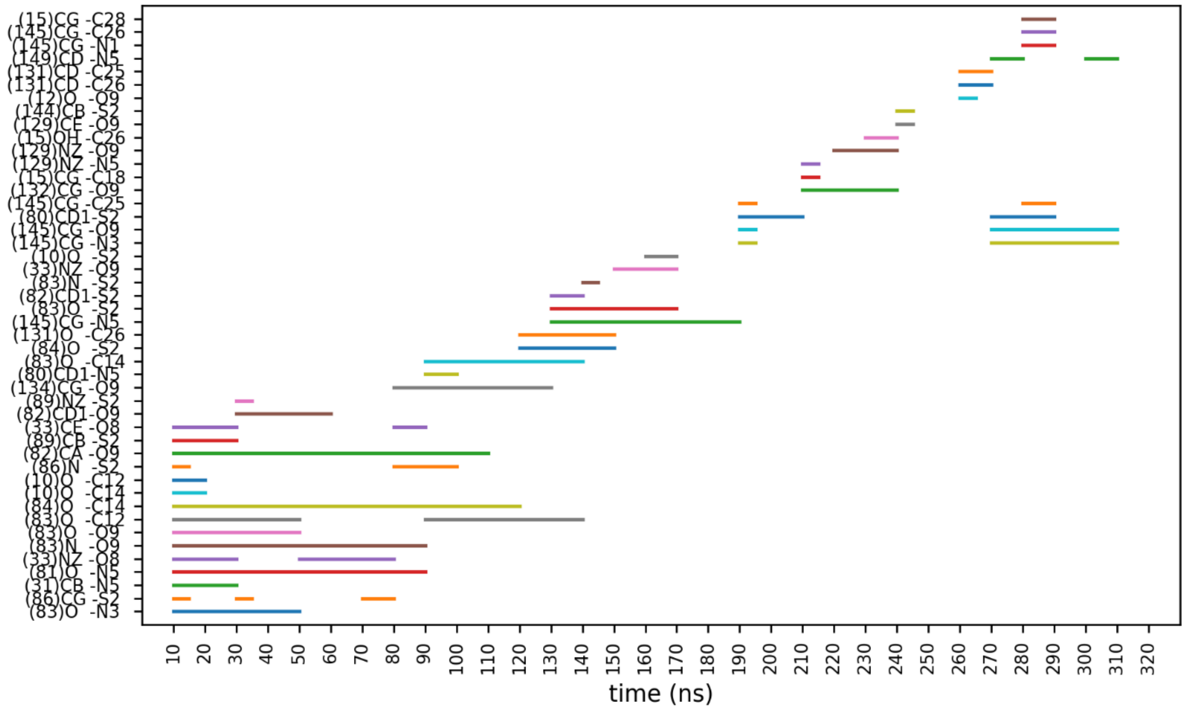

Figure S3.IV. Distances used for the main CV along the trajectory for 62K replica 1.

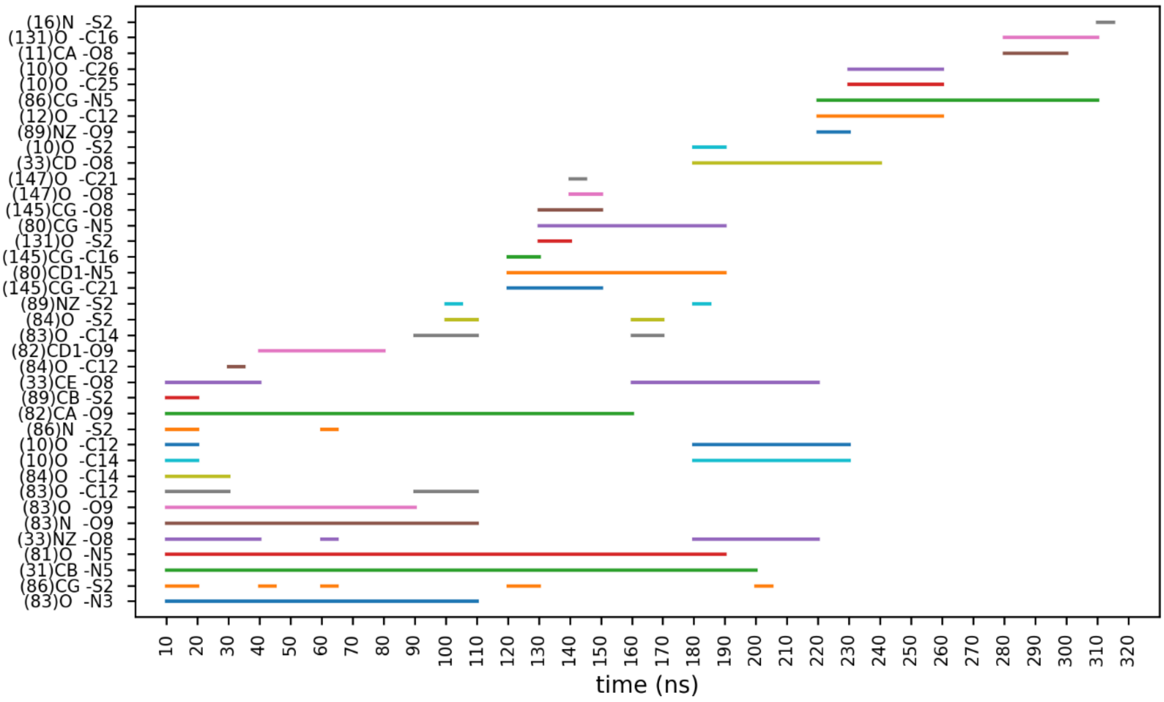

Figure S3.V. Distances used for the main CV along the trajectory for 62K replica 2.

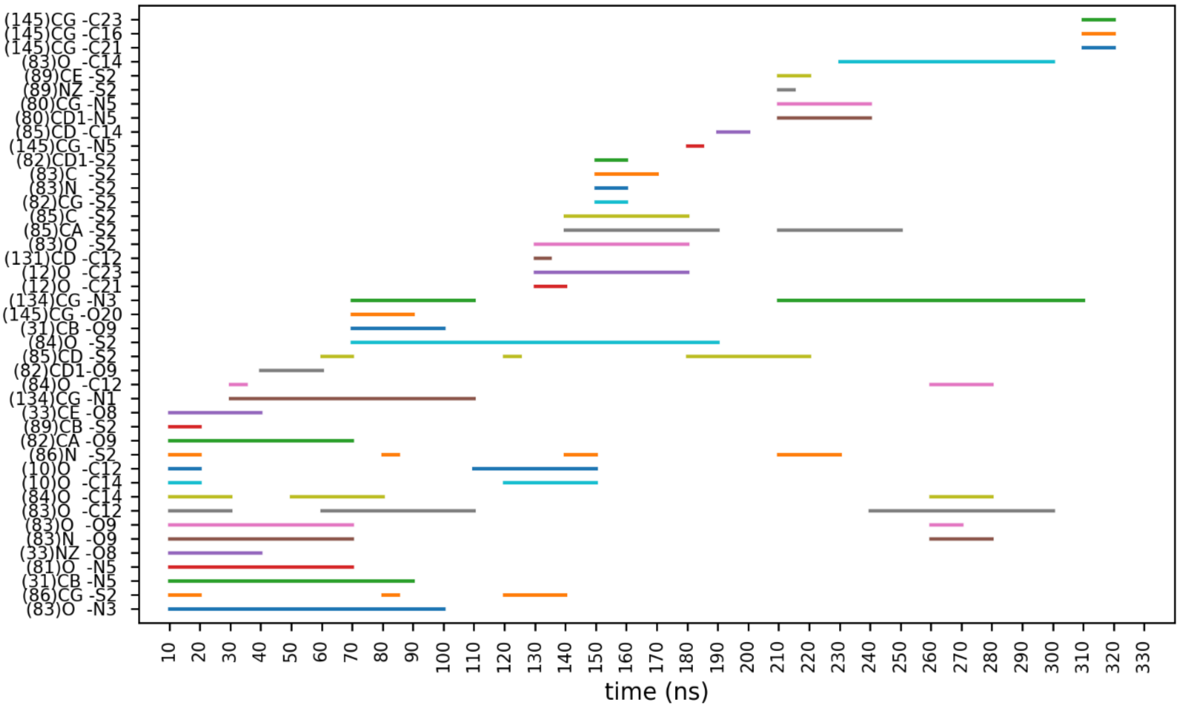

Figure S3.VI. Distances used for the main CV along the trajectory for 62K replica 3.

4. MLTSA flowchart

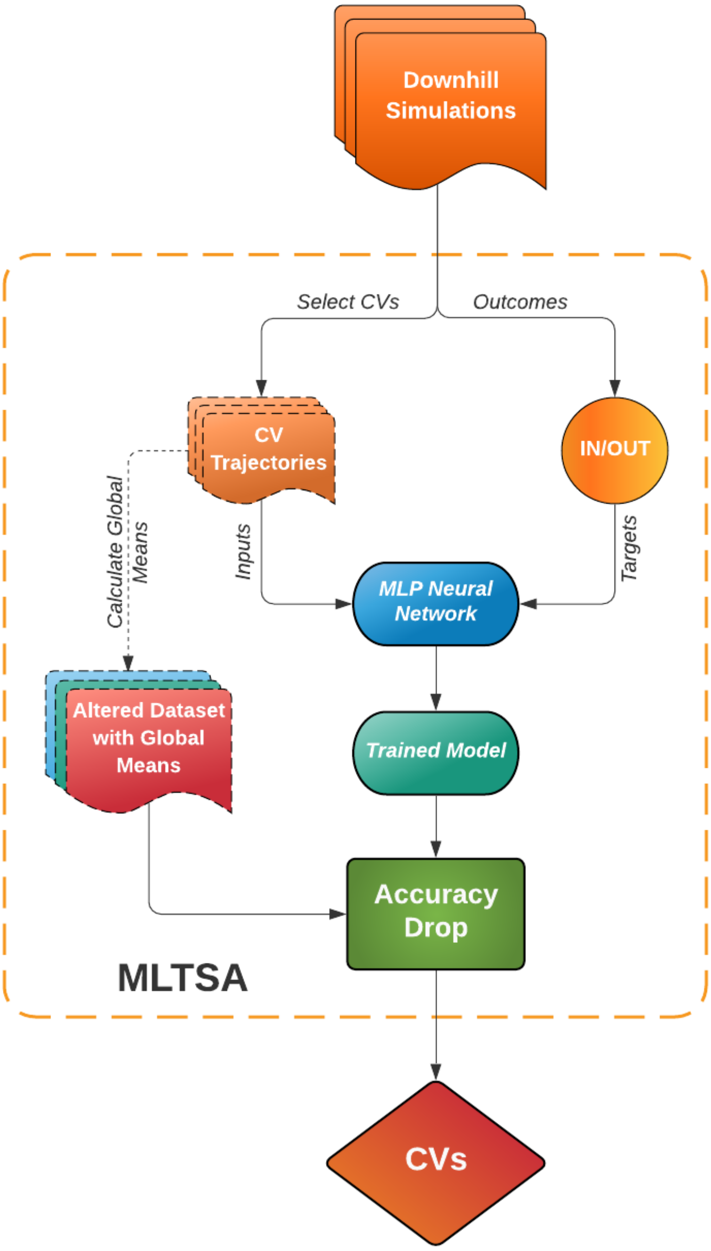

Figure S4. Flowchart of the Machine Learning Transition State Analysis (MLTSA) process. This approach uses data from the downhill unbiased simulations initiated near the TS to train a neural network on the trajectory frames using selected CVs. The model then predicts the ligand's outcome (In/Out). After successful training, the original input data (orange) is altered one CV at a time and replaced by a constant value (Global Mean), thus producing an altered dataset (Red/Green/Blue). This is used to re-predict the outcomes on the already trained model (Sea Green) and pinpoint the main accuracy drops. This allows identifying the most relevant CVs by assessing the accuracy drop.

5. Classification and Input Features for Machine Learning

Analytical model system

Two types of 1D potentials were used: single-well (SW, y_{sw}) potentials and double-well (DW, y_{dw}) potentials, defined by the equation:

$$y = k_1$$

where k_1 = 100, μ = ½, σ = 0.01, and k_2= 0 for a SW potential or k_2= 1 for a DW potential.

To generate trajectories along these model 1D free energy profiles, we used the 1D Brownian Overdamped Langevin Equation:

$$\frac{dx(t)}{dt} = \frac{y(x(t))}{\gamma(x(t))} + \sqrt{\frac{1}{\gamma(x(t))}\eta(t)}$$

where $\gamma = 0.01$is constant along *x* and $\eta(t)$ is a number randomly sampled from a normal (Gaussian) distribution centred at 0 and the spread is 1.0.

Using the trajectories generated, we defined input features ($y_{feature}$) by combining the coordinates along two different potentials (y_{pot}), either SW or DW using $y_{feature} = \alpha y_{pot1} + (1-\alpha)y_{pot2}$. We generated 180 features for both a dataset with 24 SW potentials and 1 single DW potential which decides the IN/OUT state, and for a dataset with 20 SW potentials and 5 DW potentials, having among them the decisive DW potential as well.

Table S1.I. Table containing the feature numbers for the correlated features, the values of the mixing coefficients (α_1) for the selected DW potential, and the second SW potential used for the linear combination. Coefficients for all other uncorrelated input features are not shown.

Feature Number	α_1	SW
5	**0.575**	*11*
21	**0.475**	*10*
25	**0.535**	*21*
42	**0.673**	*19*
48	**0.403**	*23*
55	**0.840**	*18*
87	**0.656**	*1*
90	**0.348**	*4*
119	**0.858**	*12*
136	**0.355**	*9*
167	**0.899**	*3*

Table S1.II. Table containing the feature numbers for the correlated features, the values of the mixing coefficients (α_1) for the selected DW potential, and the second SW or one of the five DW potentials used for the linear combination. Coefficients for all other uncorrelated input features are not shown.

Feature Number	α_1	Potential
4	**0.366**	*DW-4*
8	**0.767**	*SW-16*
22	**0.507**	*SW-6*
25	**0.880**	*SW-7*
35	**0.867**	*SW-12*
50	**0.452**	*DW-3*
62	**0.432**	*SW-18*
71	**0.321**	*SW-1*
114	**0.761**	*DW-14*
131	**0.511**	*SW-15*
137	**0.359**	*SW-24*
165	**0.390**	*SW-23*
167	**0.476**	*DW-2*
175	**0.349**	*SW-8*
177	**0.269**	*SW-20*

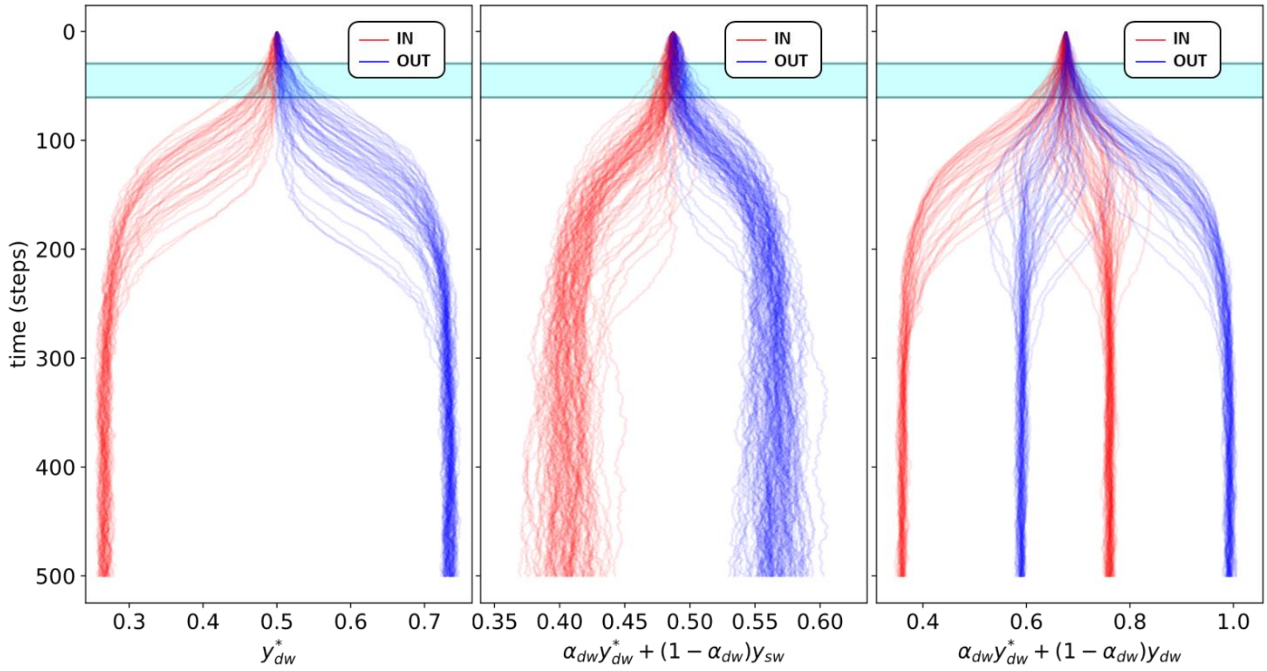

Figure S5 Left: Plot of the trajectory coordinates across different simulations depending on the classified outcome (IN-red OUT-blue) for the decisive DW potential. Middle: Plot of one correlated feature for different simulations after the linear combination between the decisive DW and a SW potential, this plot corresponds to feature 136 of the 1DW dataset, with a linear coefficient of α = 0.355. Right: Plot of a correlated feature which is combined with the decisive DW potential and another DW potential, the line colour depends on the classified outcome. In this case this corresponds to feature 4 from the 5 DW dataset, the decisive DW (marked with *) has a linear coefficient of α = 0.366. In shaded cyan the region selected for the ML training (from the 30th step to the 60th).

CDK2 system data pre-processing

The classification of the ligand in the bound position (IN) and unbound position (OUT) is calculated by analyzing the last 250 ps of the downhill trajectories. For each frame we extract and sum two key distances between the ligand and the protein (see Table S2.I) and average these for all the frames of the last 250 ps. If the sum of these distances is below a given IN-threshold the trajectory is classified as IN, if the value is above the OUT-threshold then is classified as OUT (see Table S2.I). Table S2.II presents all the interatomic distances used in the ML inputs.

Table S2.I. Key distances used to automate the IN/OUT labelling of the 5 ns-long downhill trajectories. These are used to create a dataset suitable for the ML algorithm to learn the classification with the selected CVs as inputs (X) and the labels IN/OUT as targets (Y).

SYSTEM	DISTANCES	IN-THRESHOLD	OUT-THRESHOLD
**3SW4**	LIG(N9)-LEU83(O) - LIG(N7)-LEU83(N)	10	12
**4FKW**	LIG(N3)-LEU83(O) - LIG(O9)-LEU83(N)	11	13

Table S2.II. List of all interatomic distances for heavy atoms between the ligand and the protein within 6 Å at the selected TS structure. In addition, three intra-residue distances within the protein were also added.

**3sw4 (184 Features)**	**4fkw (181 Features)**
=============================	============================
V1=LIG(S1)-VAL64(CB)	V1=LIG(N1)-ILE10(CB)
V2=LIG(S1)-VAL64(CG1)	V2=LIG(N1)-ILE10(CG1)
V3=LIG(S1)-VAL64(CG2)	V3=LIG(N1)-ILE10(CD1)
V4=LIG(S1)-PHE80(CB)	V4=LIG(N1)-GLN131(O)
V5=LIG(S1)-PHE80(CG)	V5=LIG(N1)-LEU134(CG)
V6=LIG(S1)-PHE80(CD1)	V6=LIG(N1)-LEU134(CD1)
V7=LIG(S1)-PHE80(CE1)	V7=LIG(N1)-LEU134(CD2)
V8=LIG(S1)-PHE80(CZ)	V8=LIG(S2)-ILE10(CG2)
V9=LIG(S1)-PHE80(CE2)	V9=LIG(N3)-ILE10(CB)
V10=LIG(S1)-PHE80(CD2)	V10=LIG(N3)-ILE10(CG1)
V11=LIG(S1)-LEU134(CD1)	V11=LIG(N3)-ILE10(CD1)
V12=LIG(S1)-ALA144(CB)	V12=LIG(N3)-GLN85(O)
V13=LIG(C2)-ILE10(CB)	V13=LIG(N3)-ASP86(CB)
V14=LIG(C2)-ILE10(CG2)	V14=LIG(N3)-LEU134(CG)
V15=LIG(C2)-ILE10(CD1)	V15=LIG(N3)-LEU134(CD1)
V16=LIG(C2)-VAL18(CB)	V16=LIG(N3)-LEU134(CD2)
V17=LIG(C2)-VAL18(CG1)	V17=LIG(N4)-ILE10(CA)
V18=LIG(C2)-VAL18(CG2)	V18=LIG(N4)-ILE10(CB)
V19=LIG(N3)-VAL18(CG2)	V19=LIG(N4)-ILE10(CG2)
V20=LIG(N3)-LYS33(CE)	V20=LIG(N5)-ILE10(CD1)
V21=LIG(N3)-LYS33(NZ)	V21=LIG(N5)-VAL18(CG2)
V22=LIG(N3)-PHE80(CG)	V22=LIG(N5)-ALA31(CB)
V23=LIG(N3)-PHE80(CD1)	V23=LIG(N5)-LEU134(CD1)
V24=LIG(N3)-PHE80(CE1)	V24=LIG(N5)-LEU134(CD2)
V25=LIG(N3)-PHE80(CZ)	V25=LIG(N5)-ALA144(CB)
V26=LIG(N3)-LEU134(CD1)	V26=LIG(C6)-LYS129(CD)
V27=LIG(N3)-ALA144(CB)	V27=LIG(C6)-LYS129(CE)
V28=LIG(N3)-ASP145(OD2)	V28=LIG(C6)-LYS129(NZ)
V29=LIG(C4)-VAL18(CB)	V29=LIG(C6)-GLN131(CB)
V30=LIG(C4)-VAL18(CG1)	V30=LIG(C6)-GLN131(CG)
V31=LIG(C4)-VAL18(CG2)	V31=LIG(C7)-ILE10(O)
V32=LIG(C4)-LEU134(CD1)	V32=LIG(C7)-GLY11(N)
V33=LIG(N5)-LYS33(CE)	V33=LIG(C7)-GLY11(CA)
V34=LIG(N5)-LYS33(NZ)	V34=LIG(C7)-GLY11(C)
V35=LIG(N5)-VAL64(CG1)	V35=LIG(C7)-GLU12(N)
V36=LIG(N5)-PHE80(CB)	V36=LIG(C7)-GLN131(CB)
V37=LIG(N5)-PHE80(CG)	V37=LIG(O8)-VAL18(CG1)
V38=LIG(N5)-PHE80(CD1)	V38=LIG(O8)-ASP145(CB)
V39=LIG(N5)-PHE80(CE1)	V39=LIG(O8)-ASP145(CG)
V40=LIG(N5)-PHE80(CZ)	V40=LIG(O8)-ASP145(OD2)
V41=LIG(N5)-PHE80(CE2)	V41=LIG(O9)-ILE10(CD1)
V42=LIG(N5)-PHE80(CD2)	V42=LIG(O9)-ALA31(CB)
V43=LIG(N5)-ALA144(CA)	V43=LIG(O9)-GLU81(O)
V44=LIG(N5)-ALA144(CB)	V44=LIG(O9)-LEU134(CG)
V45=LIG(N5)-ASP145(N)	V45=LIG(O9)-LEU134(CD1)
V46=LIG(N5)-ASP145(OD2)	V46=LIG(O9)-LEU134(CD2)
V47=LIG(C6)-LYS33(CE)	V47=LIG(O10)-HIE84(O)
V48=LIG(C6)-VAL64(CG1)	V48=LIG(O11)-ASP86(OD2)
V49=LIG(C6)-VAL64(CG2)	V49=LIG(O11)-LYS89(CG)
V50=LIG(C6)-PHE80(CB)	V50=LIG(O11)-LYS89(CD)
V51=LIG(C6)-PHE80(CG)	V51=LIG(O11)-LYS89(CE)
V52=LIG(C6)-PHE80(CD1)	V52=LIG(C12)-ILE10(CB)
V53=LIG(C6)-PHE80(CE1)	V53=LIG(C12)-ILE10(CG2)
V54=LIG(C6)-PHE80(CZ)	V54=LIG(C12)-ILE10(CG1)
V55=LIG(C6)-PHE80(CE2)	V55=LIG(C12)-ILE10(CD1)
V56=LIG(C6)-PHE80(CD2)	V56=LIG(C12)-PHE82(CE1)
V57=LIG(C6)-LEU134(CD1)	V57=LIG(C12)-LEU83(O)
V58=LIG(C6)-ALA144(CA)	V58=LIG(C12)-GLN85(N)
V59=LIG(C6)-ALA144(CB)	V59=LIG(C12)-GLN85(CA)
V60=LIG(N7)-ILE10(CG2)	V60=LIG(C12)-GLN85(C)

V61=LIG(N7)-ILE10(CD1)	V61=LIG(C12)-GLN85(O)
V62=LIG(N7)-LEU83(N)	V62=LIG(C13)-ILE10(CB)
V63=LIG(N7)-LEU83(CA)	V63=LIG(C13)-ILE10(CG2)
V64=LIG(N7)-LEU83(CB)	V64=LIG(C13)-ILE10(CG1)
V65=LIG(N7)-LEU83(C)	V65=LIG(C13)-ILE10(CD1)
V66=LIG(N7)-HIE84(N)	V66=LIG(C13)-ILE10(O)
V67=LIG(N7)-HIE84(CA)	V67=LIG(C13)-GLN85(C)
V68=LIG(N7)-HIE84(C)	V68=LIG(C13)-ASP86(N)
V69=LIG(N7)-HIE84(O)	V69=LIG(C13)-ASP86(CA)
V70=LIG(N7)-GLN85(N)	V70=LIG(C13)-ASP86(CB)
V71=LIG(N7)-GLN85(C)	V71=LIG(C13)-ASP86(CG)
V72=LIG(N7)-GLN85(O)	V72=LIG(C13)-ASP86(OD2)
V73=LIG(N7)-LEU134(CD1)	V73=LIG(C14)-ILE10(CB)
V74=LIG(N7)-LEU134(CD2)	V74=LIG(C14)-ILE10(CG2)
V75=LIG(C8)-ALA31(CB)	V75=LIG(C14)-ILE10(CD1)
V76=LIG(C8)-LEU134(CD1)	V76=LIG(C14)-PHE82(CE1)
V77=LIG(N9)-ILE10(CG2)	V77=LIG(C14)-LEU83(O)
V78=LIG(N9)-HIE84(C)	V78=LIG(C14)-HIE84(C)
V79=LIG(N9)-HIE84(O)	V79=LIG(C14)-HIE84(O)
V80=LIG(N9)-GLN85(C)	V80=LIG(C14)-GLN85(N)
V81=LIG(N9)-GLN85(O)	V81=LIG(C14)-GLN85(CA)
V82=LIG(N9)-ASP86(N)	V82=LIG(C14)-GLN85(C)
V83=LIG(N9)-ASP86(CA)	V83=LIG(C15)-ILE10(CB)
V84=LIG(N9)-LEU134(CG)	V84=LIG(C15)-ILE10(CG2)
V85=LIG(N9)-LEU134(CD1)	V85=LIG(C15)-ILE10(O)
V86=LIG(N9)-LEU134(CD2)	V86=LIG(C15)-GLN85(C)
V87=LIG(C10)-ILE10(CG2)	V87=LIG(C15)-ASP86(N)
V88=LIG(C10)-ILE10(CD1)	V88=LIG(C15)-ASP86(CB)
V89=LIG(C10)-ALA31(CB)	V89=LIG(C15)-ASP86(CG)
V90=LIG(C10)-LEU134(CG)	V90=LIG(C15)-ASP86(OD2)
V91=LIG(C10)-LEU134(CD1)	V91=LIG(C16)-VAL18(CB)
V92=LIG(C10)-LEU134(CD2)	V92=LIG(C16)-VAL18(CG1)
V93=LIG(N11)-ASP86(CG)	V93=LIG(C16)-VAL18(CG2)
V94=LIG(N11)-ASP86(OD1)	V94=LIG(C16)-ALA144(CB)
V95=LIG(N11)-ASP86(OD2)	V95=LIG(C16)-ASP145(CB)
V96=LIG(C12)-ILE10(CD1)	V96=LIG(C16)-ASP145(CG)
V97=LIG(C12)-ALA31(CB)	V97=LIG(C16)-ASP145(OD2)
V98=LIG(C12)-GLU81(O)	V98=LIG(C17)-VAL18(CB)
V99=LIG(C12)-LEU83(N)	V99=LIG(C17)-VAL18(CG1)
V100=LIG(C12)-LEU83(CA)	V100=LIG(C17)-VAL18(CG2)
V101=LIG(C12)-LEU83(CB)	V101=LIG(C17)-ALA144(CB)
V102=LIG(C12)-LEU83(CG)	V102=LIG(C17)-ASP145(CB)
V103=LIG(C12)-LEU134(CD1)	V103=LIG(C18)-VAL18(CB)
V104=LIG(N13)-ILE10(CG2)	V104=LIG(C18)-VAL18(CG1)
V105=LIG(N13)-ILE10(CD1)	V105=LIG(C18)-VAL18(CG2)
V106=LIG(N13)-LEU134(CG)	V106=LIG(C18)-GLN131(O)
V107=LIG(N13)-LEU134(CD1)	V107=LIG(C18)-LEU134(CD2)
V108=LIG(N13)-LEU134(CD2)	V108=LIG(C19)-LYS129(CE)
V109=LIG(C14)-ILE10(CG2)	V109=LIG(C19)-LYS129(NZ)
V110=LIG(C14)-ILE10(CD1)	V110=LIG(C19)-GLN131(CA)
V111=LIG(C14)-ALA31(CB)	V111=LIG(C19)-GLN131(CB)
V112=LIG(C14)-GLU81(O)	V112=LIG(C19)-GLN131(CG)
V113=LIG(C14)-PHE82(CA)	V113=LIG(C19)-GLN131(C)
V114=LIG(C14)-PHE82(CD1)	V114=LIG(C19)-GLN131(O)
V115=LIG(C14)-PHE82(CE1)	V115=LIG(C19)-ASN132(N)
V116=LIG(C14)-PHE82(C)	V116=LIG(C19)-ASN132(CG)
V117=LIG(C14)-LEU83(N)	V117=LIG(C19)-ASN132(OD1)
V118=LIG(C14)-LEU83(CA)	V118=LIG(C19)-ASN132(ND2)
V119=LIG(C14)-LEU83(CB)	V119=LIG(O20)-GLN131(CB)
V120=LIG(C14)-LEU83(CG)	V120=LIG(O20)-GLN131(C)
V121=LIG(C14)-LEU83(C)	V121=LIG(O20)-GLN131(O)
V122=LIG(C14)-HIE84(N)	V122=LIG(O20)-ASN132(N)
V123=LIG(C14)-HIE84(CA)	V123=LIG(O20)-ASN132(CA)
V124=LIG(C14)-HIE84(C)	V124=LIG(O20)-ASN132(CG)
V125=LIG(C14)-HIE84(O)	V125=LIG(O20)-ASN132(OD1)
V126=LIG(C14)-LEU134(CD1)	V126=LIG(O20)-ASN132(ND2)
V127=LIG(C15)-ILE10(CG2)	V127=LIG(C21)-VAL18(CG1)
V128=LIG(C15)-ILE10(CD1)	V128=LIG(C21)-ASN132(ND2)
V129=LIG(C15)-HIE84(O)	V129=LIG(C21)-ASP145(CB)
V130=LIG(C15)-GLN85(O)	V130=LIG(C21)-ASP145(OD2)
V131=LIG(C15)-LEU134(CG)	V131=LIG(C22)-ILE10(CB)

V132=LIG(C15)-LEU134(CD1)	V132=LIG(C22)-ILE10(CG1)
V133=LIG(C15)-LEU134(CD2)	V133=LIG(C22)-ILE10(CD1)
V134=LIG(C16)-ILE10(CG2)	V134=LIG(C22)-LEU83(O)
V135=LIG(C16)-GLN85(O)	V135=LIG(C22)-GLN85(C)
V136=LIG(C16)-ASP86(CA)	V136=LIG(C22)-GLN85(O)
V137=LIG(C16)-ASP86(CB)	V137=LIG(C22)-ASP86(N)
V138=LIG(C16)-LEU134(CG)	V138=LIG(C22)-ASP86(CB)
V139=LIG(C16)-LEU134(CD2)	V139=LIG(C22)-ASP86(OD2)
V140=LIG(C17)-ILE10(CG2)	V140=LIG(C22)-LEU134(CD2)
V141=LIG(C17)-GLN131(O)	V141=LIG(C23)-VAL18(CB)
V142=LIG(C17)-LEU134(CG)	V142=LIG(C23)-VAL18(CG1)
V143=LIG(C17)-LEU134(CD1)	V143=LIG(C23)-VAL18(CG2)
V144=LIG(C17)-LEU134(CD2)	V144=LIG(C23)-ASP145(CB)
V145=LIG(C18)-ILE10(CG2)	V145=LIG(C24)-ILE10(CB)
V146=LIG(C18)-GLN131(O)	V146=LIG(C24)-ILE10(CG2)
V147=LIG(C18)-LEU134(CD2)	V147=LIG(C24)-GLN85(CA)
V148=LIG(C19)-ILE10(CG2)	V148=LIG(C24)-GLN85(C)
V149=LIG(C19)-ILE10(O)	V149=LIG(C24)-ASP86(N)
V150=LIG(C19)-ASP86(CB)	V150=LIG(C24)-ASP86(OD2)
V151=LIG(C19)-ASP86(CG)	V151=LIG(C25)-ILE10(CG1)
V152=LIG(C19)-ASP86(OD1)	V152=LIG(C25)-ILE10(CD1)
V153=LIG(C19)-ASP86(OD2)	V153=LIG(C25)-VAL18(CG2)
V154=LIG(C20)-LYS88(CB)	V154=LIG(C25)-ALA31(CB)
V155=LIG(C20)-LYS88(CG)	V155=LIG(C25)-LEU134(CG)
V156=LIG(C20)-GLN131(CA)	V156=LIG(C25)-LEU134(CD1)
V157=LIG(C20)-GLN131(CB)	V157=LIG(C25)-LEU134(CD2)
V158=LIG(C20)-GLN131(CG)	V158=LIG(C26)-ILE10(CG1)
V159=LIG(C20)-GLN131(C)	V159=LIG(C26)-ILE10(CD1)
V160=LIG(C20)-GLN131(O)	V160=LIG(C26)-VAL18(CG2)
V161=LIG(C21)-ILE10(O)	V161=LIG(C26)-LEU134(CG)
V162=LIG(C21)-GLY11(CA)	V162=LIG(C26)-LEU134(CD1)
V163=LIG(C21)-ASP86(OD1)	V163=LIG(C26)-LEU134(CD2)
V164=LIG(C22)-ILE10(CG2)	V164=LIG(C27)-VAL18(CB)
V165=LIG(C22)-ILE10(O)	V165=LIG(C27)-VAL18(CG1)
V166=LIG(C22)-ASP86(CA)	V166=LIG(C27)-VAL18(CG2)
V167=LIG(C22)-ASP86(CB)	V167=LIG(C27)-LEU134(CD2)
V168=LIG(C22)-ASP86(CG)	V168=LIG(C27)-ALA144(CB)
V169=LIG(C22)-ASP86(OD1)	V169=LIG(C28)-ILE10(CG1)
V170=LIG(C22)-ASP86(OD2)	V170=LIG(C28)-ILE10(CD1)
V171=LIG(C23)-ILE10(CG2)	V171=LIG(C28)-VAL18(CB)
V172=LIG(C23)-ILE10(O)	V172=LIG(C28)-VAL18(CG1)
V173=LIG(C23)-GLN85(C)	V173=LIG(C28)-VAL18(CG2)
V174=LIG(C23)-GLN85(O)	V174=LIG(C28)-LEU134(CD2)
V175=LIG(C23)-ASP86(N)	V175=LIG(C28)-ALA144(CB)
V176=LIG(C23)-ASP86(CA)	V176=LIG(C29)-LYS129(CE)
V177=LIG(C23)-ASP86(CB)	V177=LIG(C29)-LYS129(NZ)
V178=LIG(C23)-ASP86(CG)	V178=LIG(C29)-GLN131(CB)
V179=LIG(C23)-ASP86(OD1)	V179=LEU(CA)-LYS20(C)
V180=LIG(C23)-ASP86(OD2)	V180=GLN(C)-VAL18(C)
V181=LIG(C23)-LEU134(CD2)	V181=LEU(C)-1011.086(ILE)
V182=LEU(CA)-LYS20(C)	
V183=GLN(C)-VAL18(C)	
V184=LEU(C)-1011.086(ILE)	

6. ML Training Results and Feature Analysis

Analytical model system

We generated 180 independent trajectories of 500 steps each and for each trajectory frame we calculated the 180 input features. We split the dataset into a training set (70% of all trajectories) and a test set (30% of all trajectories) for the ML training. In addition, we run additional 50 independent simulations for validation of the extent of any overfitting. We determined the ML prediction accuracy at different times by varying total number of frames (i.e., simulation intervals) and starting from different initial frames throughout the trajectories.

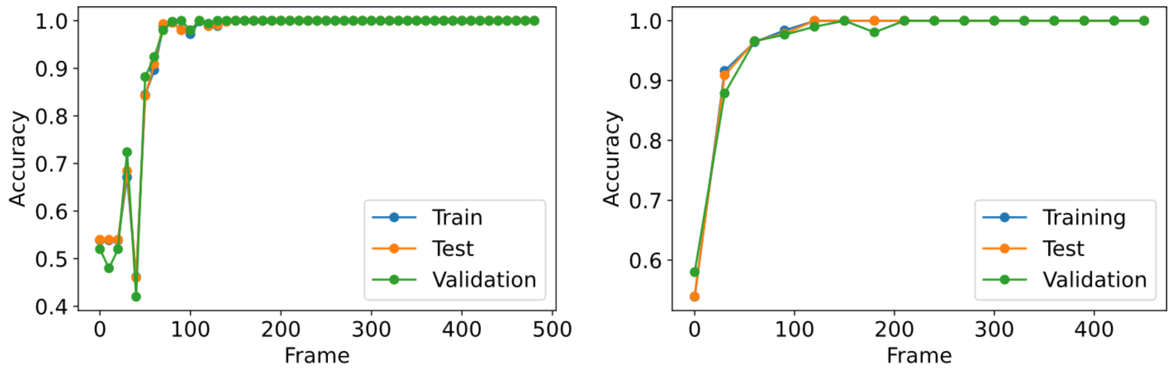

Figure S6. Accuracy of the Multi-Layer Perceptron (MLP) prediction for the dataset with 1 DW and 24 SW potentials at different starting times using 10 frame intervals (left) and 30 frame intervals (right).

For the result that a time range of 30 steps starting from the 30th step (from the 30th to 60th step) to be satisfactory for this approach. Thus, we started to train on that time range 100 models for both GBDT and MLP to proceed with our feature analysis. Some average values for accuracy and training epochs are shown in the following table:

Table S3. Table containing the average accuracies (for training, test and validation) and number of epochs used for training of GBDT and MLP methods over the 100 independent replicates of our procedure, for both types of datasets (1 DW and the 5 DW potentials) tested.

	**⟨Training Accuracy (%)⟩**	**⟨Test Accuracy (%)⟩**	**⟨Validation Accuracy (%)⟩**	**⟨Epochs⟩**
GBDT (1DW)	100.00	99.72	91.45	500
MLP (1DW)	94.83	94.73	93.04	204
GBDT (5DW)	100.00	99.80	91.64	500
MLP (5DW)	91.85	91.83	89.32	311

Analytical model with 5 - Double Well potentials MLTSA vs GBDT

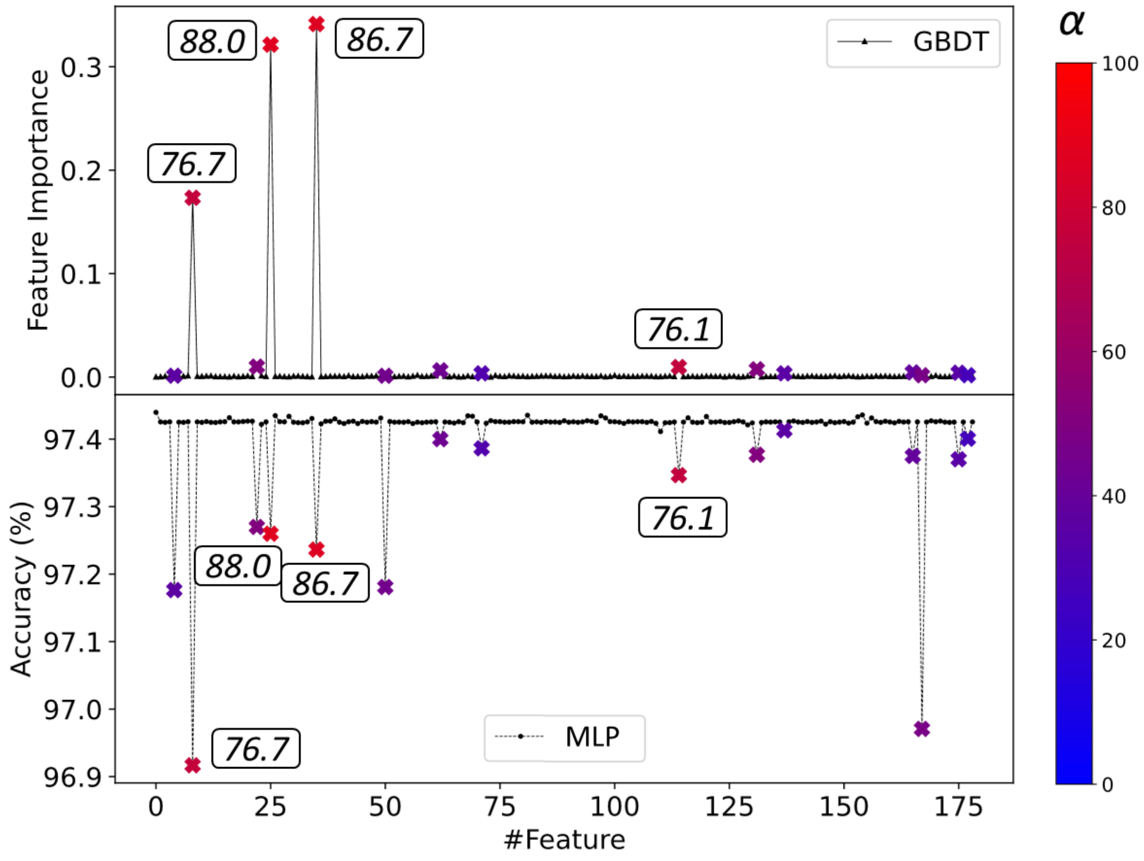

Figure S6. Comparison between GBDT (top) and MLTSA (bottom) feature analysis methods and the captured correlated features on the 5DW dataset for the analytical model. From blue (0%) to red (100%), the correlated features are marked depending on the level of correlation. The top four features are labelled. The baseline symbols (black) in the top plot show the feature importance values for each feature which are close to 0 when irrelevant. The baseline symbols (black) at the bottom plot show the accuracy of the trained data when each feature is altered with its global mean across simulations.

CDK2 System

Details of the trained models during the MLTSA using 0.1, 0.15, 0.3, 0.5, 0.75, 1, 1.5, 3, and the full 5 ns length of the downhill trajectories for each system (4fkw and 3sw4) are listed below. In addition to testing the different lengths of trajectories, the percentage of data to use from the latter end of the trajectory at each time frame (i.e., the 50% latter end of 0.1 ns would correspond to data from 0.05 ns to 0.1 ns) was also tested. The number of simulations available and the number of epochs until convergence for each model are also listed, as well as their accuracy on a set of independent simulations (Validation set). This set is comprised of the 25% of the available data from 4fkw and 3sw4 having 35 and 37 simulations to test the accuracy, respectively.

Table S4.I. The table below comprises the details of the models tested on 4fkw data as well as their accuracy on the validation set. The first column corresponds to the time frame of trajectory data used from the beginning. The data column corresponds to the percentage of latter simulation time used to train each model. The third column has the number of epochs until convergence of the model and the last column shows the accuracy on the validation set.

System		Simulations	
4fkw		139	
**Time (ns)**	**Data**	**Epochs**	**Accuracy**
**0.1**	***5%***	198	48%
	10%	142	48%
	25%	180	47%
	50%	190	47%
**0.15**	***5%***	166	45%
	10%	211	49%
	25%	223	47%
	50%	221	45%
**0.3**	***5%***	174	63%
	10%	165	63%
	25%	236	59%
	50%	205	54%
**0.5**	***5%***	173	53%
	10%	210	55%
	25%	295	46%
	50%	358	55%
**0.75**	***5%***	179	45%
	10%	112	55%
	25%	304	53%
	50%	206	52%
**1**	***5%***	105	52%
	10%	170	50%
	25%	248	55%
	50%	333	54%
**1.5**	***5%***	131	61%
	10%	195	63%
	25%	338	63%
	50%	354	62%
**3**	***5%***	148	81%
	10%	110	81%
	25%	151	79%
	50%	201	74%
**5**	***5%***	19	100%
	10%	18	99%
	25%	22	93%
	50%	73	87%

Table S4.II. The table below comprise the details of the models tested on 3sw4 data as well as their accuracy on the validation set. The first column corresponds to the time frame of trajectory data used from the beginning. The data column corresponds to the percentage of latter simulation time used to train each model. The third column has the number of epochs until convergence of the model and the last column shows the accuracy on the validation set.

System		Simulations	
3sw4		148	
**Time (ns)**	**Data**	**Epochs**	**Accuracy**
**0.1**	***5%***	192	57%
	10%	310	63%
	25%	239	53%
	50%	194	53%
**0.15**	***5%***	279	67%
	10%	215	68%
	25%	175	71%
	50%	293	70%
**0.3**	***5%***	130	57%
	10%	123	66%
	25%	293	53%
	50%	353	55%
**0.5**	***5%***	236	62%
	10%	210	62%
	25%	333	63%
	50%	191	63%
**0.75**	***5%***	231	66%
	10%	249	69%
	25%	320	65%
	50%	217	62%
**1**	***5%***	292	62%
	10%	220	60%
	25%	249	63%
	50%	229	66%
**1.5**	***5%***	229	67%
	10%	285	71%
	25%	277	62%
	50%	189	62%
**3**	***5%***	246	82%
	10%	342	75%
	25%	204	73%
	50%	153	73%
**5**	***5%***	34	100%
	10%	38	99%
	25%	103	97%
	50%	83	87%

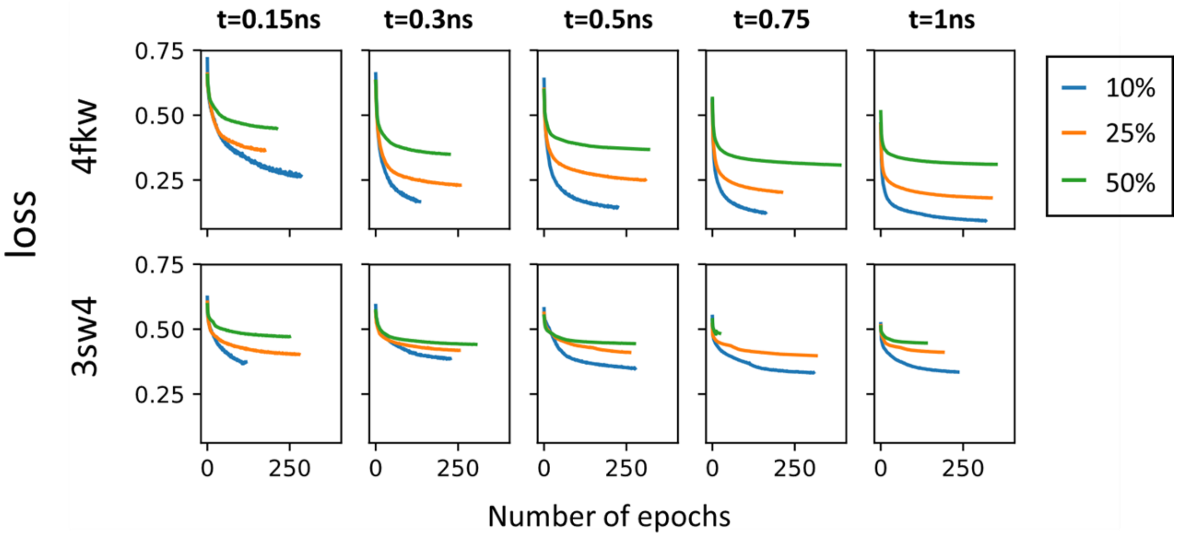

Figure S7. Plots of the loss function evolution through the training epochs at different time frames with different percentages of data from the end for both 3sw4 (18K ligand) and 4fkw (62K ligand) CDK2 systems.

To understand the relationship between the accuracy of predictions and the data used to make those predictions, we trained the MLP with several different datasets. As described in the main text, each trajectory provided a set of distances from the simulated trajectory at particular timeframes, and each dataset was made up of a set of such timeframe elements. The trainings used different timeframes of the trajectories: at 0.3, 0.5, 0.75, 1, 1.5, 3 and 5 ns. For each of these datasets we calculated the accuracy of the predictions for each of the three systems. The models provide good accuracy from the very initial frames of the simulations. For example, at 0.1 ns we have an accuracy of 79.5% for ligand 18K and 83.6% for 62K.

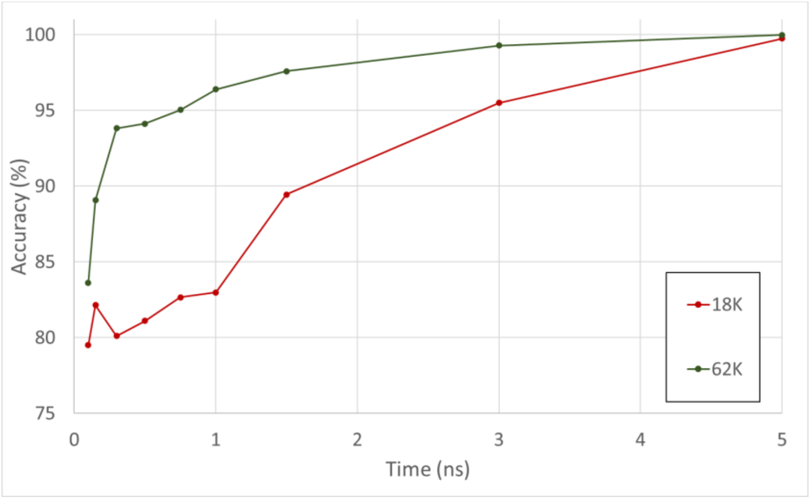

Figure S8. ML accuracy prediction at different time frames using MLP for 18K in red and 62K in green.

7. Free Energy Profiles

For each system, we performed three independent replicas. The PMF is plotted along the string windows. For each replica, the number of distances included in the string depends on the unbinding trajectory. The number of distances used in each system are given in Table S2. (Fig. S2.I-S2.VI above for details).

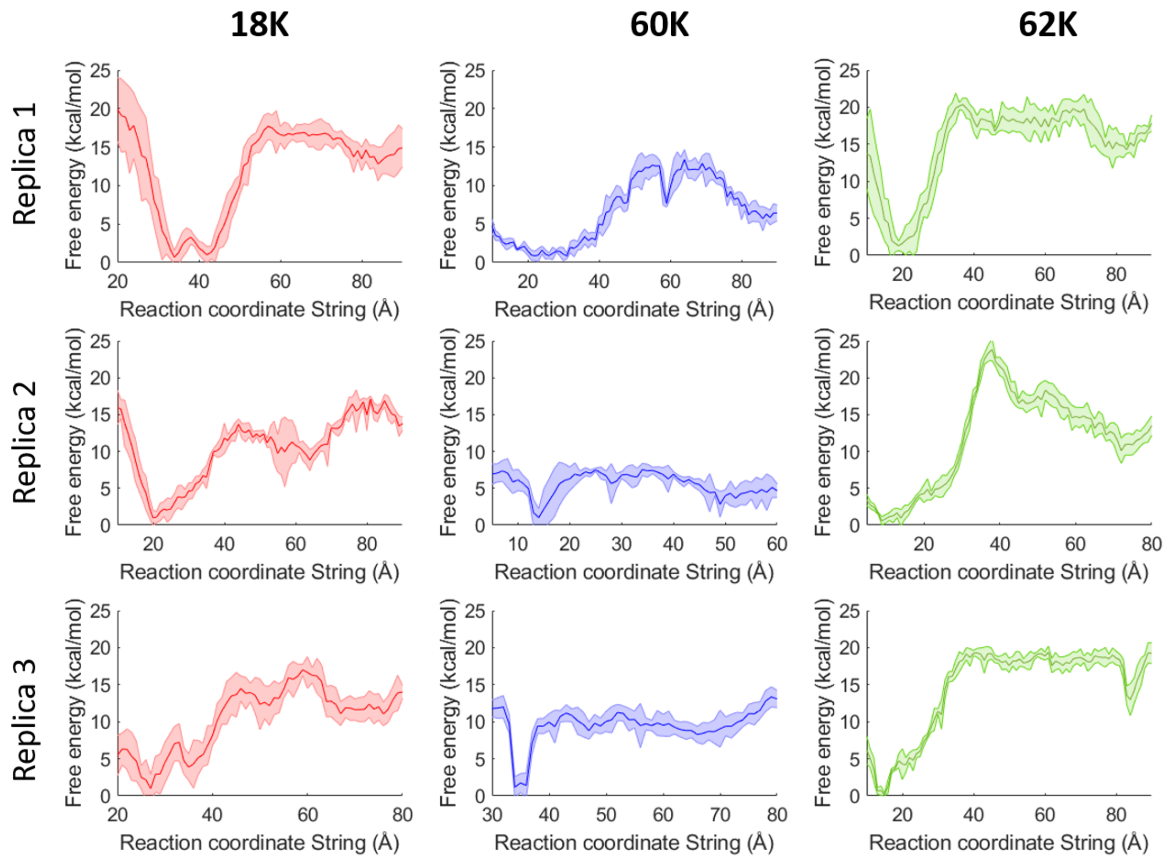

Figure S9. PMF of the unbinding path for 18K, 60K and 62K. The standard error shown as shaded area are obtained by dividing the full dataset into 4 subgroups.

Table S5. Number of distances included in the RC for each replica.

System	Number of distances
18K Replica 1	21
18K Replica 2	38
18K Replica 3	33
62K Replica 1	46
62K Replica 2	38
62K Replica 3	43

8. 60K/4FKU system

An additional ligand was tested with our unbinding approach, an oxindole carboxylic acid derivative (60K) based on the 4fku structure (Fig. S10). The unbinding procedure was carried out as described for the other ligands. After performing the string calculations for the 4fku system, 60K presented a change in conformation, more specifically, a cis-trans conversion of the hydrazineyl N=C bond (Fig. S11). This conformational change would only be expected at very high energy costs, and it is a combined artifact of the force field and the biasing procedure. The Z (cis) to E (trans) conversion allowed the 60K ligand to unbind with a significantly lower free energy barrier than its analogue, 4fkw (Figs. S12 and S13). They both share a dihedral angle (ϕ), which corresponds to this transformation, defined between atoms N6-N9-C14-C16 for 60K and N1-N3-C25-C26 for 62K (Fig. S12). On one hand, this is partly due to the initially strong constraints from the string method that can be corrected in the future. On the other hand, this is also due to the too low energy of the trans form and the too low barrier for the isomerisation as compared to the DFT calculations (Fig. S13). Tas a result, the final unbinding free energy barrier (Fig. S9, middle, blue) is ~10 kcal/mol lower than the experimental (20.01 (±0.12) kcal mol^{-1}) value for all three replicas (9.96 (±1.5) kcal mol^{-1}).

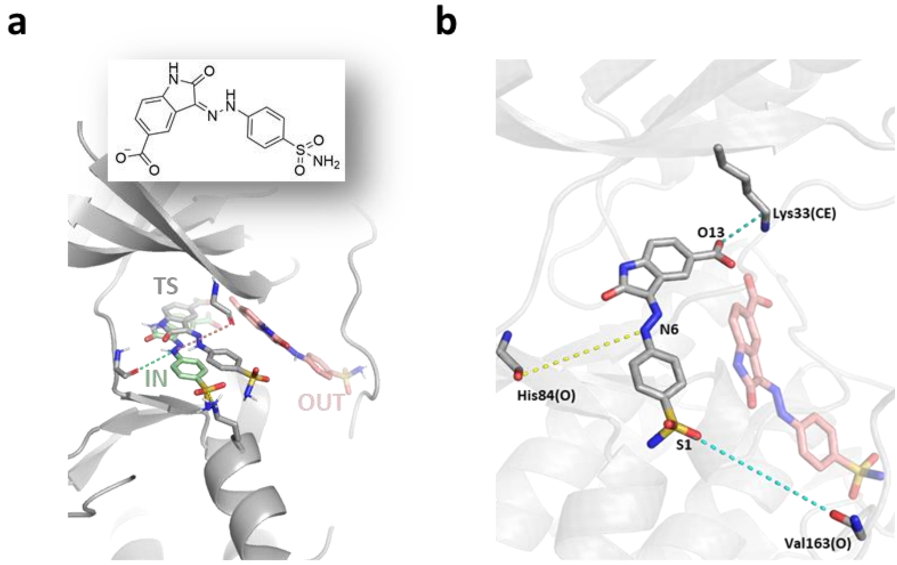

Figure S10. Left (a): CDK2 bound to 60K, the chemical structure of the ligand oxindole carboxylic acid derivative is drawn in the inset. Bound state (IN) originated from PDB structure 4fku. Structural details of the ATP pocket are shown with the ligand in the bound state (green), unbound (red) and transition state (grey). Right (b): common CVs obtained from the unbinding replicas of 60K, representative distances are shown in dashed lines (yellow: interaction from the initial structure, cyan: interaction found during the unbinding trajectory), red sticks represent the ligand when it is outside the pocket. The displayed distances appear in all three replicas for 60K.

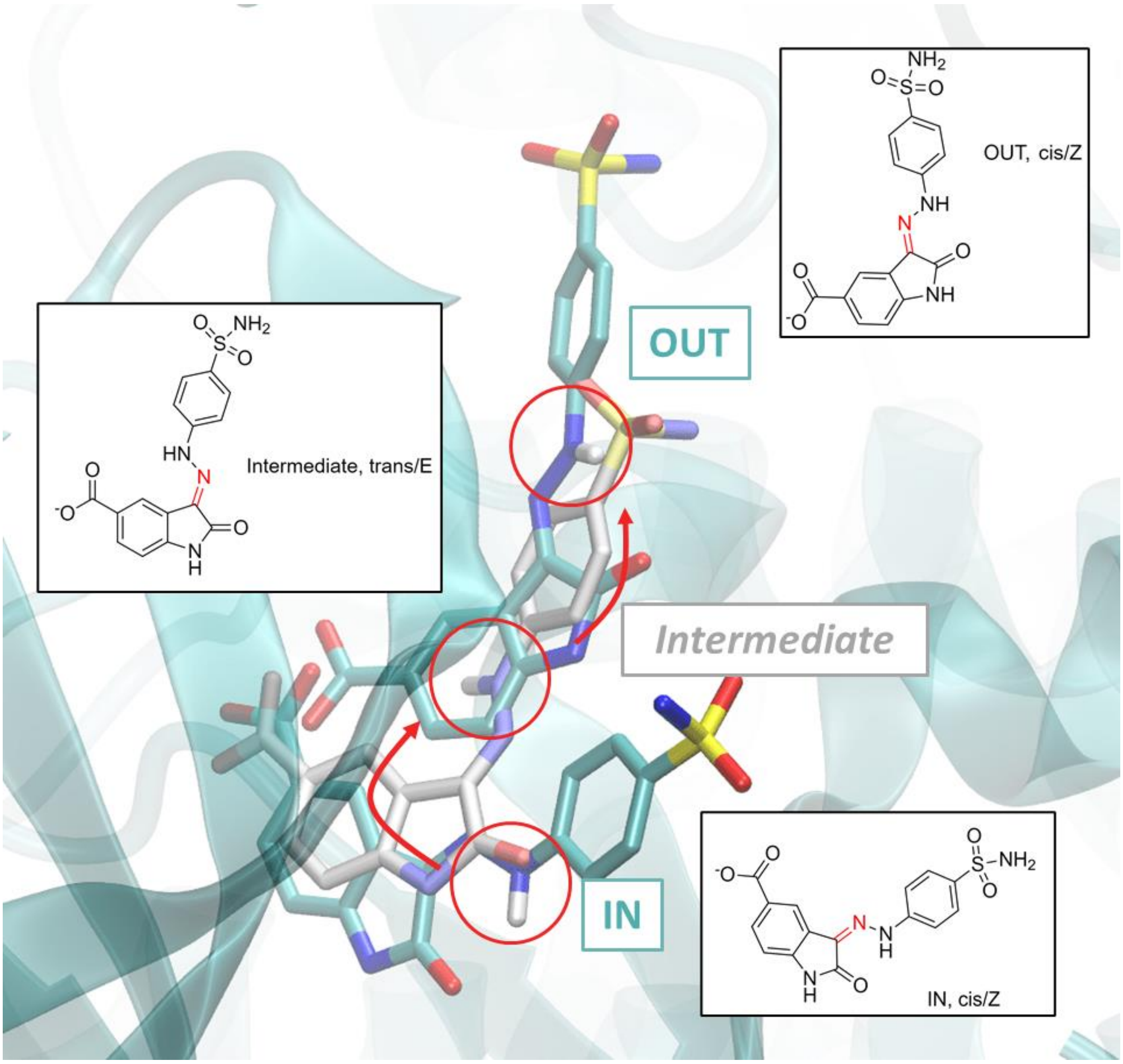

Figure S11. 60K ligand structures within the CDK2 binding pocket from three different umbrella windows portraying the cis-trans conversion through the unbinding pathway from IN (cis/Z, red circle) via the intermediate (trans/E, red circle, white sticks) to OUT (cis/Z, red circle) structures.

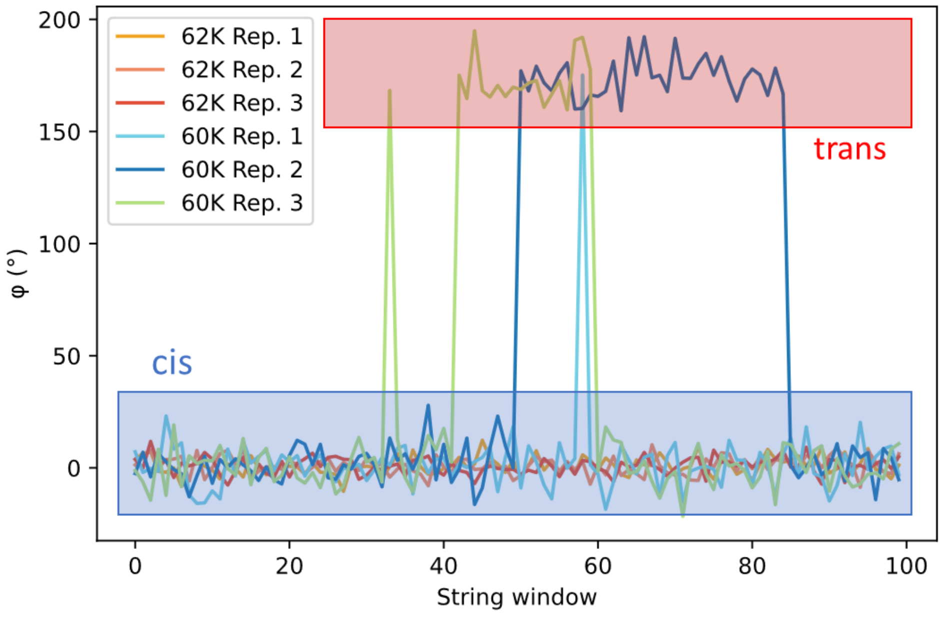

Figure S12. Values of the dihedral angles (ϕ) for both 4FKU (60K) and 4FKW (62K) throughout the umbrella sampling windows of all three replicas. ϕ is defined as the dihedral angle between atoms N6-N9-C14-C16 for 60K and C25-C26-N1-N3 for 62K. The conformations at ϕ~0° correspond to the cis isomers and at ϕ~180° correspond to the trans isomers.

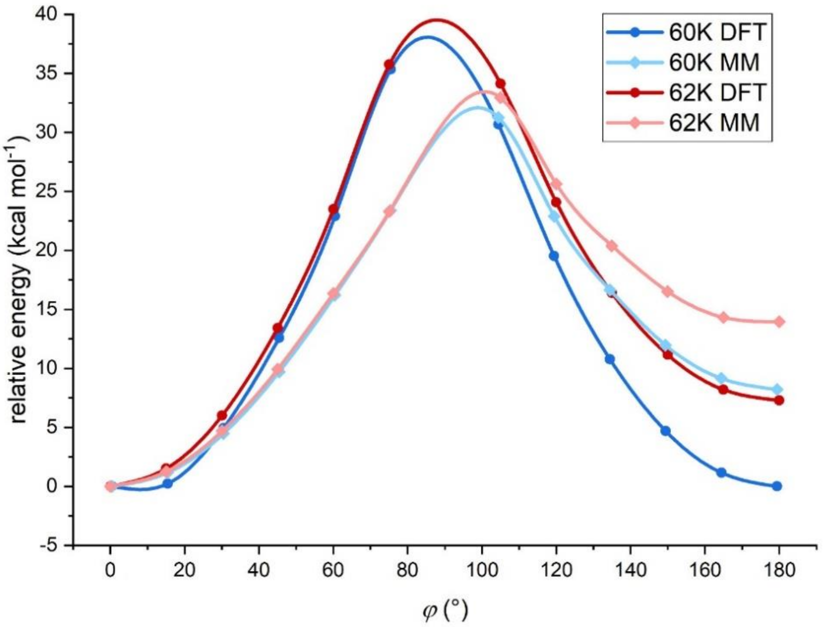

Figure S13. Relative energy values for 60K and 62K calculated at DFT (dark blue and dark red circles, respectively) and MM (light blue and pink squares, respectively) levels of theory for the cis-trans interconversion based on DFT optimized geometries along ϕ. The dihedral angle (ϕ) is defined between atoms N6-N9-C14-C16 for 60K and N1-N3-C25-C26 for 62K. The rotational barriers are lower in the force field (MM) than calculated at the DFT level. Note that the MM relative energy of the trans isomer with respect to the cis for 62K is about 10 kcal/mol higher than for 60K, contributing to the different behavior observed between the two similar ligands.

9. Validation of the ML Analysis

Figures S14.I and S14.II compare the results of our training against a simple binary classification model which attempts to classify the outcome as IN/OUT based on the CV values at a specific time (0.15, 0.3 and 0.5 ns). The dots show the CV values (as a sum of two key distances from Table S2.I) and are colored according to their outcome, red as OUT and green as IN. We then calculate the accuracy of the binary prediction at different thresholds represented by the black bars to obtain the highest possible accuracy using a single cutoff value (blue arrow). We compare these with the values obtained from the MLP (blue data, top of Figs. S14.I-II) and the GBDT (yellow data, top of Figs. S14.I-II).

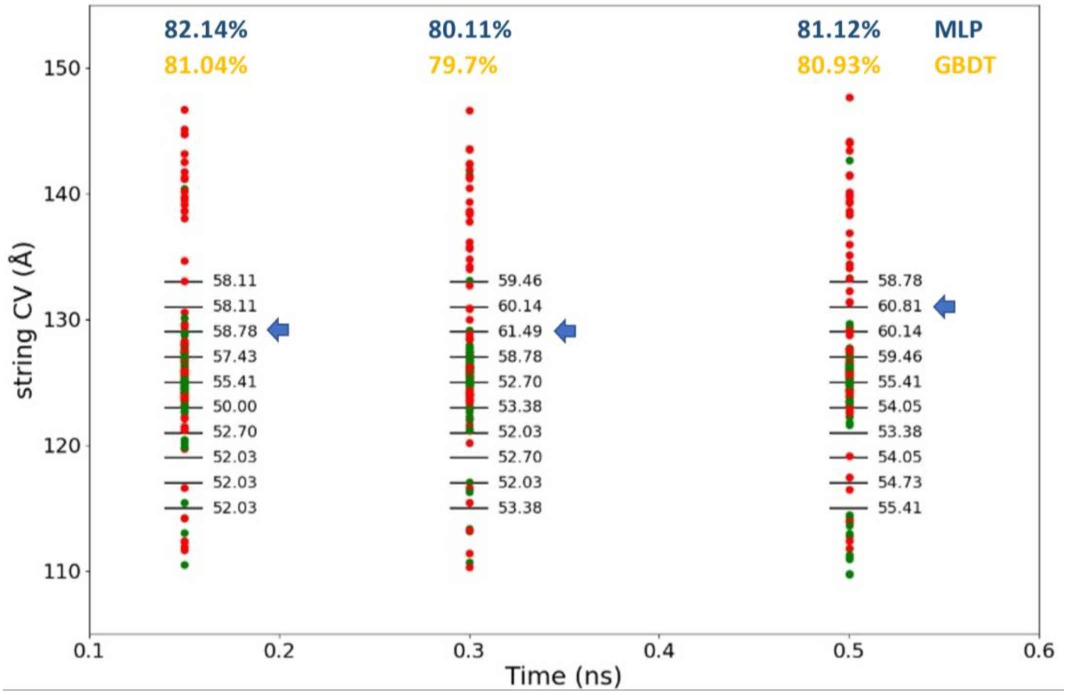

Figure S14.I. Comparison of the accuracy obtained from our MLTSA training (blue data) and GBDT (yellow data) with a simple binary classification model for ligand 18K at 0.15, 0.3 and 0.5 ns. Data points corresponding to different trajectories show the actual value of the string CV for IN (green) and OUT (red) trajectories.

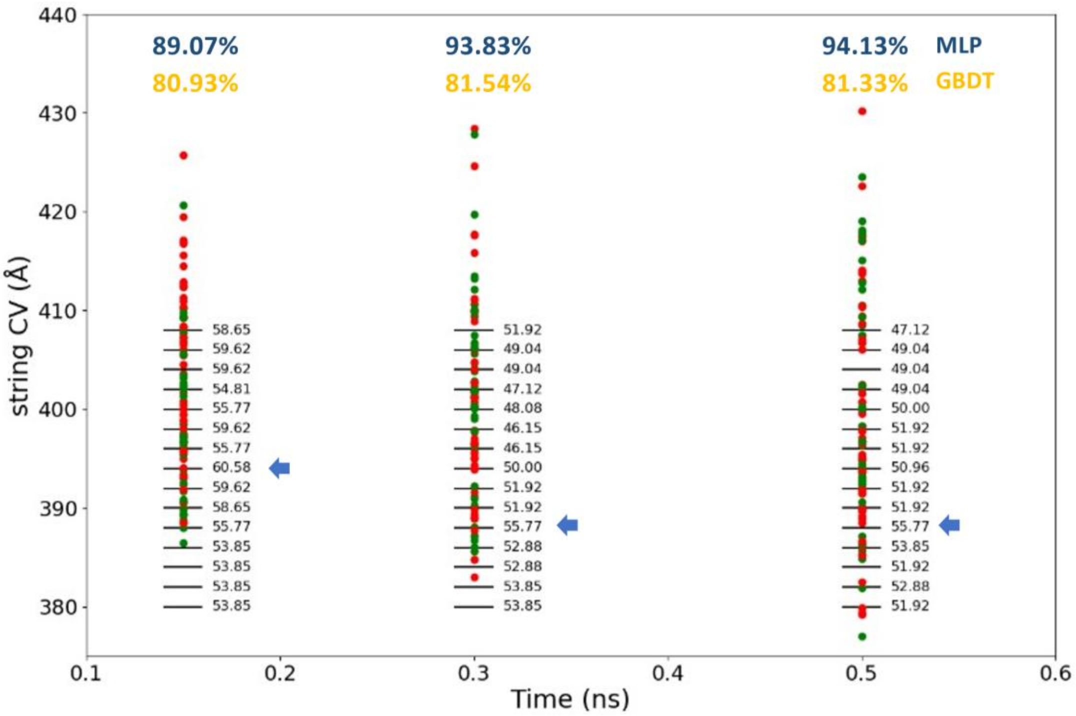

Figure S14.VII. Comparison of the accuracy obtained from our MLTSA training (blue data) and GBDT (yellow data) with a simple binary classification model for ligand 62K at 0.15, 0.3 and 0.5 ns. Data points corresponding to different trajectories show the actual value of the string CV for IN (green) and OUT (red) trajectories.

10. Gradient Boosting Decision Trees

We used GBDT as an alternative approach to the MLP. The model was trained using the same amount of data fed for the MLP. We compared the results obtained from the MLTSA against the feature importances given by the GBDT. Overall, features resulting important from the MLTSA are also present in the GBDT, however, depending on the system we analyzed, additional important features were also detected from the GBDT's important features. This suggests that the more complex non-linear behavior might lead to different performances for GBDT and the MLP as compared to the analytical model system.

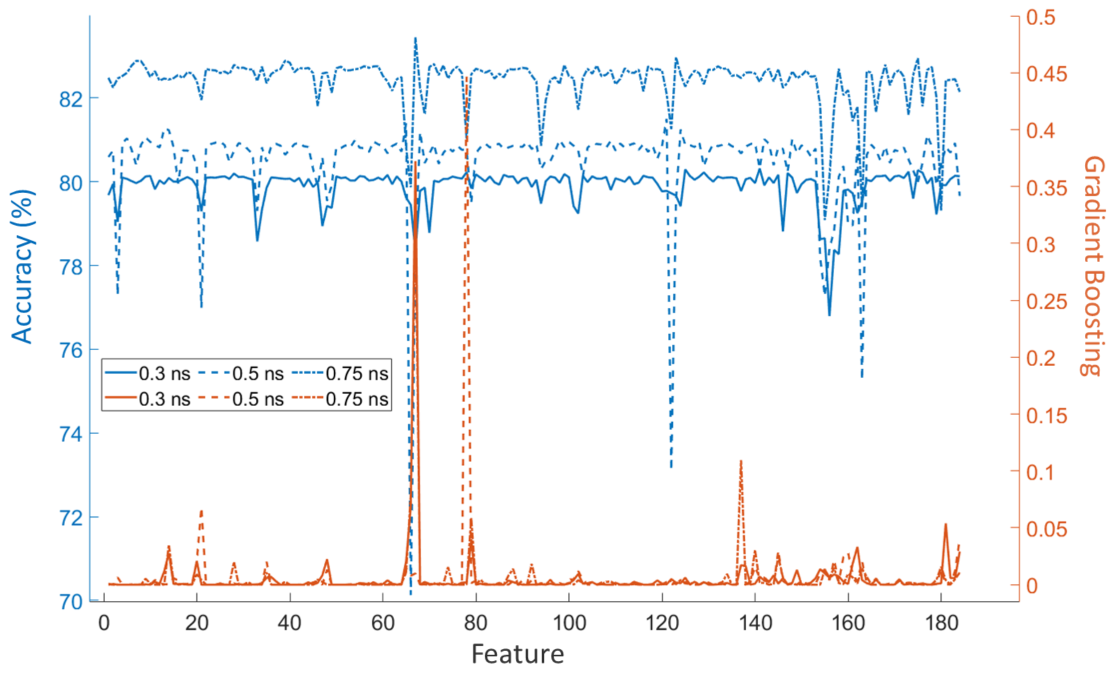

Figure S15.VIII. Comparison between GBDT feature importance (orange) and MLTSA accuracy drops (blue) at different times for the three systems for ligand 18K.

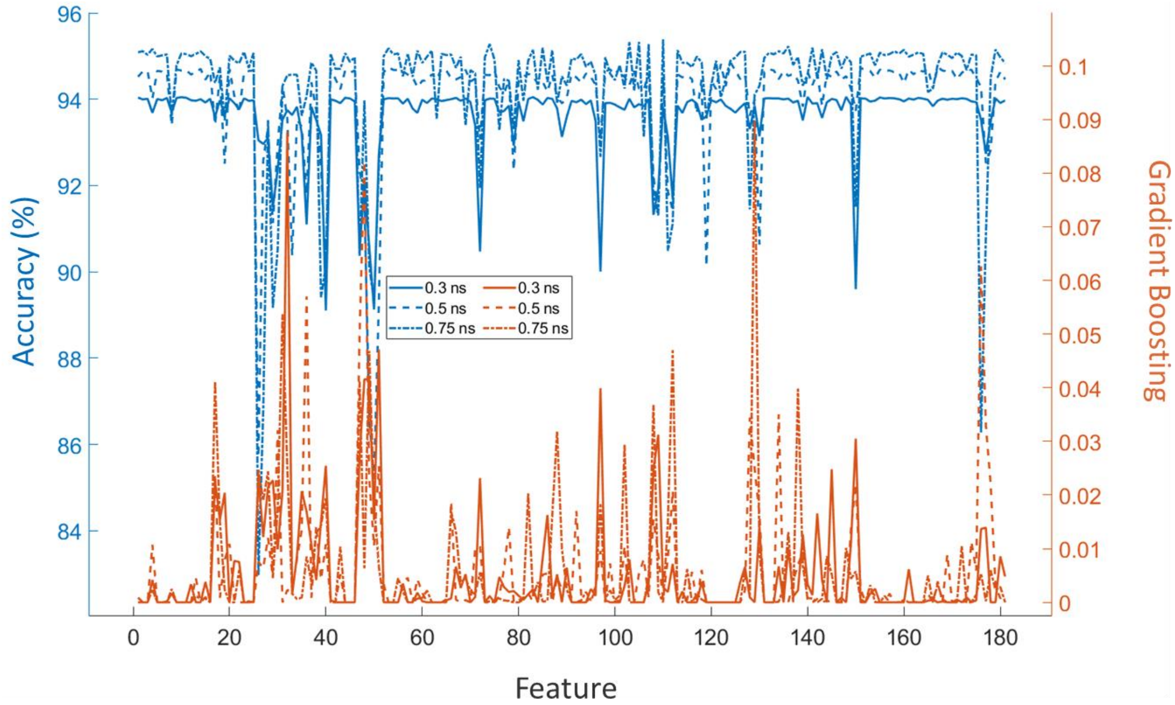

Figure S15.IX. Comparison between GBDT feature importance (orange) and MLTSA accuracy drops (blue) at different times for the three systems for ligand 62K.

11. Additional resources

Animated trajectories

Animated GIF files showing the string trajectories for the three systems (3sw4, 4fku and 4fkw) of all replicas are available at the GitHub repository:

- https://github.com/pedrojuanbj/MLTSA-V1

Software package

A Python package of the analytical MLTSA example and corresponding Python code is accessible under the Python Package Index (PyPi) database:

- https://pypi.org/project/MLTSA/

Jupyter Notebook examples

Fully annotated *Jupyter Notebook* examples on how to apply the MLTSA approach for the analytical model among others, are available under the "*MLTSA_examples*" folder on our GitHub repository as well as when installing our Python package.

12. References

1. Maier, J. A. *et al.* ff14SB: Improving the Accuracy of Protein Side Chain and Backbone Parameters from ff99SB. *J. Chem. Theory Comput.* **11**, 3696–3713 (2015).
2. Wang, J., Wolf, R. M., Caldwell, J. W., Kollman, P. A. & Case, D. A. Development and testing of a general Amber force field. *J. Comput. Chem.* **25**, 1157–1174 (2004).
3. Weigend, F. & Ahlrichs, R. Balanced basis sets of split valence, triple zeta valence and quadruple zeta valence quality for H to Rn: Design and assessment of accuracy. *Phys. Chem. Chem. Phys.* **7**, 3297–3305 (2005).
4. Chai, J. Da & Head-Gordon, M. Long-range corrected hybrid density functionals with damped atom-atom dispersion corrections. *Phys. Chem. Chem. Phys.* **10**, 6615–6620 (2008).
5. Frisch, M. J. *et al.* Gaussian 09 Revision E. (2016).
6. Phillips, J. C. *et al.* Scalable molecular dynamics with NAMD. *Journal of Computational Chemistry* vol. 26 1781–1802 (2005).
7. Feller, S. E., Zhang, Y., Pastor, R. W. & Brooks, B. R. Constant pressure molecular dynamics simulation: The Langevin piston method. *J. Chem. Phys.* **103**, 4613–4621 (1995).
8. Andersen, H. C. Rattle: A "velocity" version of the shake algorithm for molecular dynamics calculations. *J. Comput. Phys.* **52**, 24–34 (1983).
9. Essmann, U. *et al.* A smooth particle mesh Ewald method. *J. Chem. Phys.* **103**, 8577 (1998).
